# An Editorial on the Special Issue ‘Hsp90 Structure, Mechanism and Disease’

**DOI:** 10.3390/biom13030547

**Published:** 2023-03-17

**Authors:** Chrisostomos Prodromou

**Affiliations:** School of Life Sciences, Biochemistry and Biomedicine, John Maynard Smith Building, University of Sussex, Falmer, Brighton BN1 9QG, UK; chris.prodromou@sussex.ac.uk

Hsp90 is known for its role in the activation of an eclectic set of regulatory and signal transduction proteins. As such, Hsp90 plays a central role in oncogenic processes [1,2]. Although the specific details of the chaperone cycle remained controversial for a significant amount of time, the development of small molecules to inhibit Hsp90 was intense, and recently, the inhibitor Pimitespib was approved against gastrointestinal stromal tumour [3]. It is now apparent that Hsp90’s role reaches beyond what we first envisaged. The scope of this ‘Special Issue’ is to bring together this diverse information to better understand Hsp90 structure, mechanism and its role in disease. The articles not only highlight the most recent advances in our understanding, but present challenges that will need to be addressed by the scientific community. In total, six manuscripts were selected as ‘Editor’s Choice’ articles.

The review by Bjorklund and Prodromou [4], an ‘Editor’s Choice’ article, discuses recent advances in our understanding of the chaperone cycle and its regulation by co-chaperones by focusing on known structural complexes of Hsp90. Similarly, the mechanism of client protein activation by the Hsp90 complex is discussed, particularly for those of steroid hormone receptors and kinases. It appears that the Hsp90 complex is able to unfold client protein, allowing for their subsequent activation.

The article by Wengert et al. [5], also selected as an ‘Editor’s Choice’ article, reviewed the impact of mitochondrial TRAP1 on the regulation of mitochondrial function. TRAP1 is often upregulated in transformed cells and contributes to the ‘hallmarks of cancer’. It was reported that TRAP1 function is regulated by a number of post-translational modifications, which differentially impact on the function of TRAP1 in normal and cancer cells. Future progress towards understanding the post-translational or ‘chaperone code’ may inform therapeutic targeting of TRAP1 in cancer.

The review of Joshi et al. [6], another ‘Editor’s Choice article, brings together work where there has been an increased appreciation of the importance of mitochondria in regulating diverse aspects of normal cell biology, cancer and neurodegenerative disease. Much remains to be learned about the regulation of mitochondrial function and the contributions provided by their proteostasis. Specifically, how TRAP1 modulates the balance between oxidative phosphorylation (OXPHOS) by the mitochondrial electron transport chain (ETC) to generate ATP and by the less efficient process of aerobic glycolysis in the cytoplasm is discussed. It appears that TRAP1 interacts with components of the ETC to modulate activity, while the loss of TRAP1 dramatically upregulates OXPHOS by association with ETC components and suppresses their activity, forcing cells to rely on glycolysis for energy. The functional significance of TRAP1 as a dimer or higher-order oligomers is also discussed. Ongoing studies will no doubt provide additional mechanistic insight into the role of TRAP1 as a component of the mitochondrial proteostasis network.

The article by Jay et al. [7] looks at the role of extracellular Hsp90 alpha (eHsp90α), which is emerging as a newly defined research topic. It appears that inducible cellular Hsp90α (eHsp90α) is a stress-responsive isoform primarily for extracellular tissue repair. Milestone findings of recent research are elegantly brought together, including how eHsp90α is hijacked by tumours for their invasion and metastasis. It is anticipated that future therapeutic targeting of eHsp90α in wound healing and tumorigenesis could be safer and more effective than a pan inhibition of Hsp90 as a whole.

The ‘Editor Choice’ review by Backe et al. [8] looks at the large co-chaperones FNIP1, FNIP2 and Tsc1 that broaden the spectrum of Hsp90 regulators. These proteins have established roles in the regulation of tumour suppressor proteins FLCN and Tsc2. The authors discuss how their co-chaperone functions may explain their previously observed behaviour in cells and how they regulate numerous Hsp90 client proteins, including oncoproteins and tumour suppressors. Furthermore, these co-chaperones enhance Hsp90 binding to inhibitory drugs. These studies provide the groundwork for future investigations of these large co-chaperones in a pathological context as well as developing the next generation of cancer therapeutics.

The article by Ziaka and van der Spuy [9] extensively reviewed the critical role of Hsp90 in retinal proteostasis in health and disease. Current evidence highlights that the inhibition of Hsp90 as a therapeutic approach is a double-edged sword in the retina, with Hsp90 inhibition both inducing the neuroprotective upregulation of the heat shock response, but also leading to the degradation of key Hsp90 client proteins that can eventually lead to ocular toxicity. Based on the current understanding of the Hsp90 association with the retina-specific clients PDE6 and GRK1, critical components of the phototransduction cascade are reviewed. These insights will be of critical importance for the development of future next-generation molecules targeting Hsp90 proteostasis, either for the treatment of systemic or retina-specific disease.

The review by Dutta et al. [10] highlighted how the major cytosolic Hsp90 chaperone of the malaria parasite, *Plasmodium falciparum* (PfHsp90), and its associated co-chaperone complexes, display key structural and functional differences compared to the human Hsp90 system. While the core co-chaperones regulating client entry, ATPase activity and Hsp90 conformation are broadly conserved in *P. falciparum*, there are some major differences, such as the expression of two p23 isoforms, and the apparent absence of a canonical Cdc37. Overall, these differences in the co-chaperone network of PfHsp90 suggest that the elucidation of Hsp90–co-chaperone interactions can greatly extend our understanding of proteostasis and lead to the identification of novel inhibitors and drug candidates.

Piper et al. [11] bring together a review using a wealth of information on the folding of myosin heads which require a UCS (Unc45A and B, Cro1, She4) domain-containing co-chaperone for their efficient and expedient functional activation. The divergent role of Unc45 is discussed, where it appears that Hsp90α operates with Unc45B whereas Hsp90β utilises Unc45A. Given that these initial findings were first reported nearly 25 years ago, the important roles of UCS proteins are brought together, raising new questions to direct research activities.

In addition to assisting protein homeostasis, Hsp90 has been shown to be involved in the promotion and maintenance of proper protein complex assembly either alone or in association with other chaperones, such as the R2TP complex. The review by Lynham and Walid [12] looks at R2TP and its role in complex assembly. However, the molecular basis of function of Hsp90-R2TP in complex assembly has yet to be determined, and this review brings together the current understanding of the function of Hsp90-R2TP in the assembly, stabilization and activity of several client protein complexes.

The review by Mankovich and Freeman [13] shows that Hsp90 interacts with a broad array of client proteins, including a variety of factors involved in protein trafficking. Despite the numerous reports demonstrating a Hsp90 connection to protein transport, our understanding on how Hsp90 contributes to this complex process remains limited. Given the immense contributions that both Hsp90 and protein trafficking have to human health, a better comprehension on how these factors intersect is merited. The review brings together the latest research findings forming a platform to expediate research in this field of study.

The review by Maiti and Picard [14], an ‘Editor’s Choice’ article, discusses the client and functional specificities of Hsp90α and Hsp90β isoforms and whether they have specific roles in cancer, neurodegeneration and aging. Beyond gaining a better fundamental understanding of why there are two distinct isoforms, recent progress in developing isoform-specific inhibitors raises the prospects of translating new knowledge into novel therapies, and evidence is presented where a pathological process is primarily supported by one particular isoform. In such cases, targeting the relevant isoform might be ‘safer’ since ‘housekeeping’ functions could still be provided by the other isoform. This view is supported by genetic evidence where yeast or mammalian cells are viable with a single isoform.

The review by van Oosten-Hawle [15] looks at the role of Hsp90 at an organismal level, where Hsp90 regulates the proteostasis cell-nonautonomously across tissues through its involvement in cross-tissue stress signaling responses. Recent advances in the field of organismal proteostasis and its regulation by Hsp90 at a tissue-specific and organismal level have major implications on how animals respond to environmental challenges and age-associated disease. Future aspects that need to be researched further will be to understand how the different tissue-specific roles of Hsp90 are integrated into an organism to regulate survival and behavioural cues with whole-organism benefits.

The ‘Editor’s Choice’ article by Haufeng [16] discusses whether proteins always fold to a free-energy minimum or whether they can be maintained by energy-consuming processes in functional conformations with elevated free energies. The common view is that chaperones accelerate the kinetics of the folding process so that substrates reach their free-energy minimum. However, Xu challenges this view by presenting a theoretical model of non-equilibrium protein folding. It answers some long-standing questions in chaperone-assisted folding, including the necessity of ATP hydrolysis and how timing in the chaperone cycle affects the folding efficiency, and raises questions around protein structure predictions based on (free) energy minimization. Xu suggests new proteomic experiments to identify proteins that depend on ATP-dependent chaperones for the maintenance of their native structures and how to test such predictions.

The study by Omkar et al. [17] explored the importance of molecular chaperones for the activation of the Exonuclease APE2. Although APE2 has recently been demonstrated to be an important player in the DNA damage response and in a variety of human pathologies including cancer, there are currently no therapeutics that target APE2. The authors demonstrate that APE2 interacts with Hsp70 and Hsp90 in both budding yeast and mammalian cells. Furthermore, the inhibition of molecular chaperones using small molecule inhibitors promoted the rapid degradation of APE2. Taken together, this work suggests a novel way to manipulate APE2 function in cancer.

A second contribution by Bjorklund et al. [18] looks at the activation of the class 2 BRAF mutant L597R. The authors discuss how BRAF addiction for Hsp90 is broken upon dimerization of the kinase. A surprising finding was that although the L597R BRAF mutant was dimeric, it possessed no kinase activity. This has profound implications as this suggests that this class 2 mutant must therefore be dependent on RAS for its activation. Consequently, it appears that Hsp90′s role, at least with BRAF, is not the direct activation, of the kinase, but to maintain an inactive kinase that may then be delivered to the membrane-bound RAS system for its activation. In the case for the L597R mutant, signaling could occur from a CRAF-L597R heterodimer. Clinically, this is important because it could determine the way such cancers are treated—by focusing on CRAF inhibition, rather than on the inactive L597R BRAF protein.

In conclusion, the ‘Special Issue’ brings together a series of review and research papers that concentrate on past and recent advances on Hsp90. It highlights its diverse role form the cytoplasm to the extracellular environment and even at the organismal level, and concentrates on the structure, mechanism and its pivotal position in disease. The articles bring together a diverse array of research findings into specific articles that will help drive research effectively towards making further progress in this fascinating research field that is Hsp90.
